# A cost-utility analysis of drug treatments in patients with HBeAg-positive chronic hepatitis B in Thailand

**DOI:** 10.1186/1472-6963-14-170

**Published:** 2014-04-14

**Authors:** Narisa Tantai, Usa Chaikledkaew, Tawesak Tanwandee, Pitsaphun Werayingyong, Yot Teerawattananon

**Affiliations:** 1 Department of Pharmacy, Faculty of Medicine, Siriraj Hospital, 2 Prannok Road, Siriraj, Bangkoknoi, Bangkok 10700, Thailand; 2 Social and Administrative Pharmacy Excellence Research (SAPER) Unit, Department of Pharmacy, Faculty of Pharmacy, Mahidol University, 447 Sri-Ayudthaya Road, Payathai, Ratchathewi, Bangkok 10400, Thailand; 3 Division of Gastroenterology, Department of Medicine, Faculty of Medicine, Siriraj Hospital, 2 Prannok Road, Siriraj, Bangkoknoi, Bangkok 10700, Thailand; 4 Health Intervention and Technology Assessment Program (HITAP), 6th floor, 6th Building, Department of Health, Ministry of Public Health, Tiwanon Road, Muang, Nonthaburi 11000, Thailand

**Keywords:** Chronic disease, Hepatitis B, Cost-utility analysis, Treatment

## Abstract

**Background:**

Only lamivudine has been included for patients with chronic hepatitis B (CHB) in the National List of Essential Drugs (NLED), a pharmaceutical reimbursement list in Thailand. There have also been no economic evaluation studies of CHB drug treatments conducted in Thailand yet. In order to fill this gap in policy research, the objective of this study was to compare the cost-utility of each drug therapy (Figure 1) with palliative care in patients with HBeAg-positive CHB.

**Methods:**

A cost-utility analysis using an economic evaluation model was performed to compare each drug treatment for HBeAg-positive CHB patients. A Markov model was used to estimate the relevant costs and health outcomes during a lifetime horizon based on a societal perspective. Direct medical costs, direct non-medical costs, and indirect costs were included, and health outcomes were denoted in life years (LYs) and quality-adjusted life years (QALYs). The results were presented as an incremental cost effectiveness ratio (ICER) in Thai baht (THB) per LY or QALY gained. One-way sensitivity and probabilistic sensitivity analyses were applied to investigate the effects of model parameter uncertainties.

**Results:**

The ICER values of providing generic lamivudine with the addition of tenofovir when drug resistance occurred, generic lamivudine with the addition of tenofovir based on the road map guideline, and tenofovir monotherapy were -14,000 (USD -467), -8,000 (USD -267) , and -5,000 (USD -167) THB per QALY gained, respectively. However, when taking into account all parameter uncertainties in the model, providing generic lamivudine with the addition of tenofovir when drug resistance occurred (78% and 75%) and tenofovir monotherapy (18% and 24%) would yield higher probabilities of being cost-effective at the societal willingness to pay thresholds of 100,000 (USD 3,333) and 300,000 (USD 10,000) THB per QALY gained in Thailand, respectively.

**Conclusions:**

Based on the policy recommendations from this study, the Thai government decided to include tenofovir into the NLED in addition to generic lamivudine which is already on the list. Moreover, the results have shown that the preferred treatment regimen involves using generic lamivudine as the first-line drug with tenofovir added if drug resistance occurs in HBeAg-positive CHB patients.

## Background

Approximately 350 million people are chronically infected with the hepatitis B virus (HBV) [1] and nearly 25% of these carriers develop serious liver diseases such as chronic hepatitis, cirrhosis, and hepatocellular carcinoma (HCC), resulting in more than one million deaths every year [1]. Chronic liver diseases and HCC associated with HBV infections are two of the most important public health problems in high-prevalence regions [2]. In particular, most Southeast Asian countries including Thailand have been classified as high prevalence areas of HBV [1].

The goal of drug treatments for chronic hepatitis B (CHB) is to improve quality of life and survival by preventing the disease from developing into cirrhosis, decompensated cirrhosis, end-stage liver disease, HCC, and death by reducing viral replication to the lowest possible level and maintaining it over the long-term. Currently, six CHB medications including both oral (i.e., lamivudine, adefovir, entecavir and telbivudine) and subcutaneous (i.e., conventional interferon and pegylated interferon) dosage forms have been licensed by the Thai Food and Drug Administration (FDA). Additionally, tenofovir – an approved drug for the treatment of HIV but not CHB – is currently being prescribed to CHB patients in 300 mg daily doses due to its high viral efficacy and low resistance rates [3,4].

Previous cost-utility analysis studies of oral CHB medications revealed that telbivudine [5] or adefovir [6,7] was more cost-effective when compared with lamivudine. In addition, most studies performed in the US [8-14], Australia [15], and Asia [16,17] demonstrated that entecavir was superior when compared with lamivudine. Another study by Buti et al. showed that tenofovir was the better cost-effective treatment when compared with entecavir, telvibudine, and adefovir [13]. Moreover, previous economic evaluation studies of subcutaneous CHB treatments indicated that interferon was not cost-effective when compared with lamivudine [18,19] but was more cost-effective when compared to lamivudine with the addition of adefovir when drug resistance occurred [20]. The combination of lamivudine and interferon would increase life expectancy and reduce the lifetime risk of cirrhosis and carcinoma [21,22]. Furthermore, pegylated interferon was more cost-effective when compared with lamivudine [21,23,24] or interferon [7]. Most economic evaluation studies of CHB treatment were carried out in the US and Europe. However, no study has ever been performed in Southeast Asian countries, including Thailand - a high prevalence area of CHB. In addition, there has also been no economic evaluation study of the combination of CHB treatments according to the current clinical practice guidelines on the management of CHB drug resistance until now.

**Figure 1:**
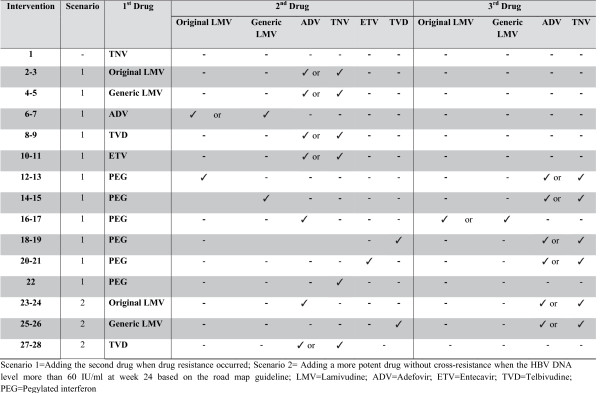
**All available interventions compared with palliative care.** Scenario 1=Adding the second drug when drug resistance occurred; Scenario 2= Adding a more potent drug without cross-resistance when the HBV DNA level more than 60 IU/ml at week 24 based on the road map guideline; LMV=Lamivudine; ADV=Adefovir; ETV=Entecavir; TVD=Telbivudine; PEG=Pegylated interferon.

When this study was conducted, only lamivudine - but not other CHB treatments with low rates of drug resistance - had been included for patients with CHB in the National List of Essential Drugs (NLED), the pharmaceutical reimbursement list referred to by three health insurance schemes which are Social Security Scheme (9% of the Thai population), Civil Servant Medical Benefit Scheme (11% of the Thai population) and Universal Coverage Scheme (80% of the Thai population), as a pharmaceutical benefit scheme in Thailand. The selection criteria for the inclusion of the NLED are safety, efficacy as well as cost-effectiveness information of drugs. The Subcommittees for Development of NLED had requested the cost-effectiveness information on CHB treatments from Health Intervention and Technology Assessment Program (HITAP), the institution responsible for appraising a wide range of health technologies including pharmaceuticals, medical devices, interventions, individual and community health promotion and prevention interventions. Therefore, the objective of this study was to compare the cost-utility of each drug therapy (Figure 1) with consideration for the management of CHB drug resistance with palliative care in patients with HBeAg-positive CHB based on a societal perspective. The results from this study would be used as the cost-effectiveness information to assist health policy makers (i.e., the Subcommittees for Development of NLED) to make policy decision whether which CHB drugs should be included in the NLED. The inclusion of CHB drugs to the NLED could have an impact on the reimbursement of CHB drugs for all HBeAg-positive CHB patients under three health insurance schemes (i.e., Social Security Scheme, Civil Servant Medical Benefit Scheme and Universal Coverage Scheme) which accounts for 100% of Thai population.

In Thailand, palliative care has been a usual care for patients with CHB, therefore it was used as a comparator in this study, since CHB-infected individuals usually develop an acute infection which may or may not result in symptoms. Those who do not exhibit symptoms and have never received hepatitis B screening test may not be aware that they have CHB until they finally develop serious liver diseases (e.g., chronic hepatitis, cirrhosis, and HCC) which consequently require palliative care. In addition, the clinical practice guidelines for the diagnosis and management of HBeAg-positive CHB - detailed in the Thailand Consensus Recommendations for Management of CHB 2009 by the former Liver Society Thailand, now known as the Thai Association for the Study of the Liver (THASL) - did not have a recommendation for the most appropriate drug to use as the standard treatment for CHB patients [25,26].

## Methods

A cost-utility analysis using a Markov model was conducted to compare the costs and health outcomes of all available drug treatments in HBeAg-positive CHB patients with palliative care; the analysis was performed using a lifetime horizon with a one-year cycle length based on a societal perspective. The study population was a hypothetical cohort of one thousand HBeAg-positive CHB patients aged at least 30 years old who required the treatment based on the following criteria: 1) patients who had detectable serum HBsAg for at least 6 months; 2) patients who had serum ALT levels 1.5 -10 times the upper limit of the normal range for at least 3 months; 3) patients who had a detectable level of serum hepatitis B viral DNA more than or equal to 20,000 IU/ml; and/or 4) patients who had evidence of CHB based on liver biopsy results. Ethical approval for this study was granted by the Institutional Review Board Committees from Mahidol University.

Since this analysis set out to compare all available CHB medications for the treatment of HBeAg-positive CHB with palliative care as a usual care and comparator in Thailand, both oral (i.e., original lamivudine, generic lamivudine, adefovir, entecavir, telbivudine and tenofovir) and subcutaneous dosage forms (i.e., pegylated interferon) were included. Among all the drugs, only tenofovir showed low drug resistance rates in the treatment of CHB [3,4] while others demonstrated high drug resistance rates. Therefore, two scenarios based on the current clinical practice guidelines on the management of CHB drug resistance were created for the study. Figure 1 presents all interventions compared with palliative care.

In the first scenario, if patients taking the original lamivudine, generic lamivudine, adefovir, entecaviror, or telbivudine encountered drug resistance, a second medication would be added to the treatment regimen based on the guidelines (10 interventions). Moreover, for CHB patients receiving pegylated interferon who failed the treatment, the second drug would be added in the third year. Then, a third drug would be added if the patient encountered drug resistance again (11 interventions).

The second scenario involved adding a more potent drug without cross-resistance when the HBV DNA level was more than 60 IU/ml at week 24 based on the roadmap guideline, which applies to only low genetic barrier drugs (i.e., lamivudine and telbivudine) (6 interventions). Thus, a total of 28 interventions (i.e., tenofovir monotherapy, 21 interventions of the first scenario and 6 interventions of the second scenario) were compared with palliative care (Figure 1).

### Model structure

**Figure 2:**
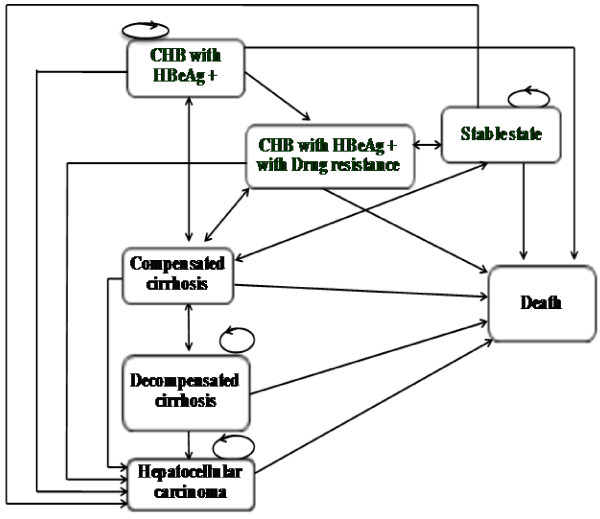
Schematic diagram of the Markov model.

Figure 2 shows the schematic diagram of the Markov model showing that all hypothetical patients aged at least 30 years old who required the treatment would start at the CHB with HBeAg-positive state. For patients receiving antiviral drugs, they would proceed to the drug resistance state if drug resistance was detected or the level of serum HBV DNA reached levels higher than 60 IU/ml. Patients receiving palliative care or those successfully treated with pegylated interferon in the first year would move to the stable state, while HBeAG-positive CHB patients – either with or without drug resistance – would also be able to transition to this state if they developed HBeAg seroconversion. In addition, patients in the stable state could also reverse to the CHB with HBeAg-positive state. HBeAg-positive CHB patients - either with or without drug resistance - and those in the stable state could progress to the compensated cirrhosis, decompensated cirrhosis, and HCC states. Patients in either the compensated or decompensated cirrhosis state could reverse to a primary state except for those with HCC, who could move to a death state only. Patients in all states could stay at the same state and could move to a death state. Both scenarios had the same model assumptions, which were: 1) the efficacy of generic lamivudine was the same as that of the original lamivudine, and 2) each treatment had differences in the seroconversion and resistance rates.

### Transitional probabilities

Table 1 demonstrates all the parameters used in the model. Due to the limitation of data, especially the clinical efficacy of CHB drugs in Thailand, these parameter values were obtained from internationally published literature [27,28]. However, we performed an indirect comparison meta-analysis of these parameters which represents the highest reliable evidence [29]. The transitional probabilities of clinical efficacy in terms of HBeAg seroconversion of HBeAg-positive CHB treatment options were estimated from a systematic review and meta-analysis using a Bayesian random effects model analyzed by WinBUGS1.4 (Medical Research Council and Imperial College of Science, Technology and Medicine, United Kingdom) [30]. All other transitional probabilities were obtained from published articles in Thailand and other countries [31-44]. In addition, the mortality rates of Thailand’s general population at each age were used in the analysis [45]. Time-invariant survival rates for each drug therapy were applied.

**Table 1:** Input parameters used in economic model

**Parameter**	**Distribution**	**Mean**	**SE**	**Reference**
**Yearly discount rate (%)**				
Costs and outcomes (range)		3 (0-6)		[51]
**Transitional probability baseline parameters**				
Probability of stable to CHB state	Beta	0.143	0.0650	[31]
Probability of CHB to stable state	Beta	0.056	0.0180	[32]
Probability of CHB to compensated in 1^st^-10^th^ year	Beta	0.054	0.0543	[33]
Probability of CHB to compensated in 11^th^-20^th^ year	Beta	0.134	0.1338	[33]
Probability of CHB to compensated in >20^th^ year	Beta	0.329	0.3292	[33]
Probability of CHB to HCC in 1^st^-5^th^ year	Beta	0.000	0.0000	[34]
Probability of CHB to HCC in 6t^h^-10^th^ year	Beta	0.006	0.0061	[34]
Probability of CHB to HCC in >10^th^ year	Beta	0.008	0.0081	[34]
Probability of CHB to death in 1^st^-5^th^ year	Beta	0.010	0.0102	[34]
Probability of CHB to death in 6^th^-10^th^ year	Beta	0.014	0.0144	[34]
Probability of CHB to death in >10^th^ year	Beta	0.025	0.0252	[34]
Probability of compensated to decompensated in 1^st^-3^rd^ year	Normal	0.042	0.0003	[35]
Probability of compensated to decompensated in 4^th^-5^th^ year	Normal	0.094	0.0005	[35]
Probability of compensated to decompensated in >5^th^ year	Normal	0.066	0.0003	[35]
Probability of compensated to HCC in 1^st^-3^rd^ year	Normal	0.014	0.0002	[35]
Probability of compensated to in HCC 4^th^-5^th^ year	Normal	0.036	0.0003	[35]
Probability of compensated to HCC in >5^th^ year	Normal	0.030	0.0002	[35]
Probability of compensated to death in 1^st^-3^rd^ year	Beta	0.014	0.0135	[35]
Probability of compensated to death in >3^rd^ year	Beta	0.046	0.0461	[35]
Probability of decompensated to HCC	Beta	0.035	0.0354	[36]
Probability of decompensated to death in 1^st^ year	Normal	0.260	0.0004	[37]
Probability of decompensated to death in 2^nd^ year	Normal	0.390	0.0005	[37]
Probability of decompensated to death in >2^nd^ year	Normal	0.240	0.0003	[37]
Probability of HCC to death in 1^st^ year	Beta	0.848	0.0011	[37]
Probability of HCC to death in >1^st^ year	Beta	0.920	0.0009	[37]
**Transitional probability of treatment parameters**				
Probability of CHB to compensated	Beta	0.006	0.0023	[38]
Probability of CHB to HCC	Beta	0.009	0.0045	[39]
Probability of CHB to death	Beta	0.002	0.0023	[39]
Probability of compensated to HCC	Beta	0.015	0.0034	[40]
Probability of compensated to death	Beta	0.007	0.0070	[39]
Probability of decompensated to HCC	Beta	0.035	0.0127	[41]
Probability of decompensated to death	Beta	0.126	0.0291	[42]
Probability of compensated to CHB	Beta	0.478	0.0665	[43]
Probability of HCC to death	Beta	0.034	0.0227	[44]
Relative risk of seroconversion of lamivudine	Normal	3.519	1.3707	[30]
Relative risk of seroconversion of adefovir	Normal	3.028	1.3833	[30]
Relative risk of seroconversion of telbivudine	Normal	4.286	1.4054	[30]
Relative risk of seroconversion of entecavir	Normal	3.846	1.3833	[30]
Relative risk of seroconversion of pegylated interferon	Normal	5.356	1.4987	[30]
Relative risk of seroconversion of tenofovir	Normal	4.167	1.6403	[30]
Probability of delay seroconversion of pegylated interferon	Normal	0.410	0.0489	[27]
Probability of lamivudine resistance	Beta	0.214	0.0214	[28]
Probability of adefovir resistance	Beta	0.066	0.0066	[28]
Probability of telbivudine resistance	Beta	0.089	0.0089	[28]
Probability of entecavir resistance	Beta	0.002	0.0002	[28]
Probability of tenofovir resistance	Beta	0.000	0.0000	[28]
**Annual direct medical cost**				
Cost of generic lamivudine	Gamma	1,797	180	[46]
Cost of original lamivudine	Gamma	34,871	3,487	[46]
Cost of adefovir	Gamma	70,298	7,030	[46]
Cost of telbivudine	Gamma	51,504	5,150	[46]
Cost of entecavir	Gamma	85,745	8,575	[46]
Cost of tenofovir	Gamma	15,559	1,556	[46]
Cost of pegylated interferon	Gamma	527,379	52,738	[46]
Cost of treatment of compensated cirrhosis	Gamma	81,264	81,264	[48]
Cost of treatment of decompensated cirrhosis	Gamma	125,127	125,127	[48]
Cost of treatment of HCC	Gamma	153,021	153,021	[48]
Cost of laboratory for screening (i.e., HBeAg, HBeAb)	Gamma	650	650	[47]
Cost of laboratory for pre-treatment	Gamma	3,350	3,350	[47]
Cost of laboratory for monitoring	Gamma	4,200	4,200	[47]
Cost of laboratory monitoring for pegylated interferon	Gamma	10,620	10,620	[47]
Cost of laboratory monitor for adefovir	Gamma	4,560	4,560	[47]
Cost of laboratory for post-treatment	Gamma	4,900	4,900	[47]
**Annual direct non-medical cost**				
Cost of transportation	Gamma	571	571	[54]
Cost of food	Gamma	210	210	[54]
**Annual indirect cost**				
Cost of time loss due to receiving treatment	Gamma	824	824	[49]
Cost of productivity loss of compensated cirrhosis	Gamma	48	48	[48,49]
Cost of productivity loss of decompensated cirrhosis	Gamma	627	627	[48,49]
Cost of productivity loss of HCC	Gamma	1,701	1,701	[48,49]
**Utility**				
Utility weight for CHB	Normal	0.68	0.00005	[53]
Utility weight for compensated cirrhosis	Normal	0.69	0.00016	[53]
Utility weight for decompensated cirrhosis	Normal	0.35	0.00031	[53]
Utility weight for HCC	Normal	0.38	0.00026	[53]

### Cost

Costs and health outcomes were estimated over a 70-year period in order to cover the expected lifetime horizon. The costs of the CHB state included the costs of antiviral drugs and laboratory and diagnostic tests, which were projected over a 70-year time horizon using the Markov model. For the number of antiviral drug utilization and laboratory and diagnostic tests used, these values were estimated based on the suggested recommendations of the THASL clinical practice guidelines [25,26]. The prices of antiviral drugs were obtained from the reference prices published by the Thai Ministry of Public Health’s Drug and Medical Supply Information Center (DMSIC) [46]. The unit costs of the laboratory and diagnostic tests were retrieved using the reference prices published by the Comptroller General’s Department of the Thai Ministry of Finance [47]. Furthermore, the costs of complication states such as compensated cirrhosis, decompensated cirrhosis, and HCC were obtained from a published study based in Thailand [48]. However, the costs of treatment for adverse drug events were not included in this study.

Direct non-medical costs (i.e., the costs of transportation, food, and time loss due to receiving treatment) but not direct medical costs incurred outside the hospital were included. All direct non-medical and the number of days due to sick leave were obtained from a published study [48]. As for indirect costs, these also included morbidity costs and were calculated from the productivity loss due to sick leave. It should be noted that mortality costs were excluded. Indirect costs were calculated from the number of days due to sick leave multiplied by the minimum wage rate of the Thai population obtained from the Thai Ministry of Labor’s Department of Labor Protection and Welfare [49]. All costs were converted and reported in year 2010 values using the consumer price index (CPI) [50] and all future costs were discounted at a rate of 3% [51] due to the time horizon being longer than one year. The average annual exchange rate of Thai baht (THB) to one US dollar was 30 THB in 2010 [52]. For international comparison, costs were converted to international dollars using the purchasing power parity (PPP) $ exchange rate of 1 PPP$ (2010) per 17.8 THB [53].

### Health outcomes

Health outcomes were denoted in life years (LYs) gained and quality-adjusted life years (QALYs) gained (i.e., the multiplication of LYs gained and the utility score) and the utility or quality of life scores of patients were obtained from a published study after we performed a systematic review on electronic databases (i.e., Pubmed and Cochrane databases) [54]. The health outcomes of each intervention were compared with palliative care. Future outcomes were also discounted at a rate of 3% [51]. The results were presented as an incremental cost effectiveness ratio (ICER) in Thai baht (THB) per a LY or QALY gained.

### Uncertainty analysis

A one-way sensitivity analysis and probabilistic sensitivity analysis (PSA) were conducted to examine the effect of parameter uncertainty in the model. All parameters in the one-way sensitivity analysis were varied across the range of confidence intervals. In addition, net monetary benefit (NMB) was calculated to determine the intervention which gave the maximum expected NMB for each value of the ceiling ratio (i.e., the value of society’s willingness to pay (WTP) for an intervention giving one QALY gained). In Thailand, the WTP per one QALY thresholds for the implementation of health technology and intervention based on two subcommittees - the Subcommittee for the Development of the National List of Essential Drugs and the Subcommittee for the Development of the Benefit Packages, National Health Security Office (NHSO) - are 100,000 (USD 3,333) and 300,000 (USD 10,000) THB per QALY gained (i.e., about one and three times the gross domestic product (GDP) per capita) [55]. Once the analysis was completed, the results of the PSA were presented using cost-effectiveness acceptability curves.

## Results

The total costs, LYs, QALYs, and ICER values of all treatments compared with palliative care in patients with HBeAg-positive CHB aged 30 years old and above are shown in Table 2. Interventions with negative ICER values indicate that they were more effective and had lower costs compared with palliative care. Thus, it can be seen that providing generic lamivudine and adding tenofovir when drug resistance occurred (ICER = -9,000 THB or USD -300 per LY gained or -14,000 THB or USD -467 per QALY gained) proved to be the most cost-effective option. The next best treatment regimens were generic lamivudine plus tenofovir based on the roadmap guideline (ICER = -5,000 THB or USD -167 per LY gained or -8,000 THB or USD -267 per QALY gained) and tenofovir monotherapy (ICER = -3,000 THB or USD -100 per LY gained or -5,000 THB or USD -167 per QALY gained).

**Table 2:** Total costs, LYs and QALYs of all interventions for HBeAg positive CHB patients aged 30 years old and above

**Interventions**	**Total costs (THB)**^**§**^	**LYs**	**QALYs**	**Incremental cost (THB)**	**Incremental QALYs**	**ICER per QALY gained**
**First drug (Second drug) Third drug**						
1. Generic lamivudine (tenofovir)^†^	456,000	20.87	13.66	-72,000	5.03	Dominant*
2. Generic lamivudine (tenofovir)^‡^	490,000	20.87	13.66	-38,000	5.03	Dominant*
3. Tenofovir monotherapy^†^	501,000	20.89	13.67	-26,000	5.04	Dominant*
4. Palliative care	527,000	13.13	8.63	-	-	-
5. Original lamivudine (tenofovir)^†^	937,000	20.87	13.66	409,000	5.03	81,000
6. Pegylated interferon (original lamivudine) tenofovir^†^	953,000	20.89	13.67	426,000	5.05	84,000
7. Original lamivudine (tenofovir)^‡^	971,000	20.87	13.66	444,000	5.03	88,000
8. Generic lamivudine (adefovir)^†^	982,000	20.87	13.66	454,000	5.03	90,000
9. Pegylated interferon (tenofovir)^†^	1,057,000	20.91	13.69	530,000	5.06	105,000
10. Telbivudine (tenofovir)^†^	1,091,000	20.90	13.68	564,000	5.05	112,000
11. Generic lamivudine (adefovir)^‡^	1,134,000	20.87	13.66	606,000	5.03	121,000
12. Telbivudine (tenofovir)^‡^	1,134,000	20.87	13.66	606,000	5.03	121,000
13. Pegylated interferon (original lamivudine) tenofovir^†^	1,325,000	20.89	13.67	798,000	5.05	158,000
14. Adefovir (generic lamivudine)^†^	1,364,000	20.85	13.64	837,000	5.01	167,000
15. Pegylated interferon (lamivudine) adefovir^†^	1,371,000	20.89	13.67	844,000	5.05	167,000
16. Telbivudine (adefovir)^†^	1,429,000	20.90	13.68	902,000	5.05	178,000
17. Pegylated interferon (telbivudine) tenofovir^†^	1,442,000	20.92	13.69	915,000	5.06	181,000
18. Original lamivudine (adefovir)^†^	1,463,000	20.87	13.66	936,000	5.03	186,000
19. Entecavir (tenofovir)^†^	1,519,000	20.88	13.67	991,000	5.04	197,000
20. Entecavir (adefovir)^†^	1,536,000	20.88	13.67	1,009,000	5.04	200,000
21. Adefovir (original lamivudine)^†^	1,564,000	20.85	13.64	1,037,000	5.01	207,000
22. Original lamivudine (adefovir)^‡^	1,616,000	20.87	13.66	1,088,000	5.03	216,000
23. Pegylated interferon (adefovir) generic lamivudine^†^	1,648,000	20.88	13.66	1,120,000	5.04	222,000
24. Telbivudine (adefovir)^‡^	1,657,000	20.90	13.68	1,130,000	5.05	224,000
25. Pegylated interferon (telbivudine) adefovir^†^	1,710,000	20.92	13.69	1,182,000	5.06	233,000
26. Pegylated interferon (original lamivudine) adefovir^†^	1,744,000	20.89	13.67	1,216,000	5.05	241,000
27. Pegylated interferon (entecavir) tenofovir^†^	1,771,000	20.90	13.68	1,243,000	5.05	246,000
28. Pegylated interferon (entecavir) adefovir^†^	1,785,000	20.90	13.68	1,257,000	5.05	249,000
29. Pegylated interferon (adefovir) original lamivudine^†^	1,812,000	20.88	13.66	1,284,000	5.04	255,000

^†^Scenario 1: Adding drug when drug resistance occurred; ^‡^Scenario 2: Adding drug based on the road map guideline.

§total costs are calculated in 2010 THB and rounded up to nearest 1,000 THB.

*Negative ICER due to higher effectiveness and lower costs of intervention compared with palliative care.

### Uncertainty analysis

**Figure 3:**
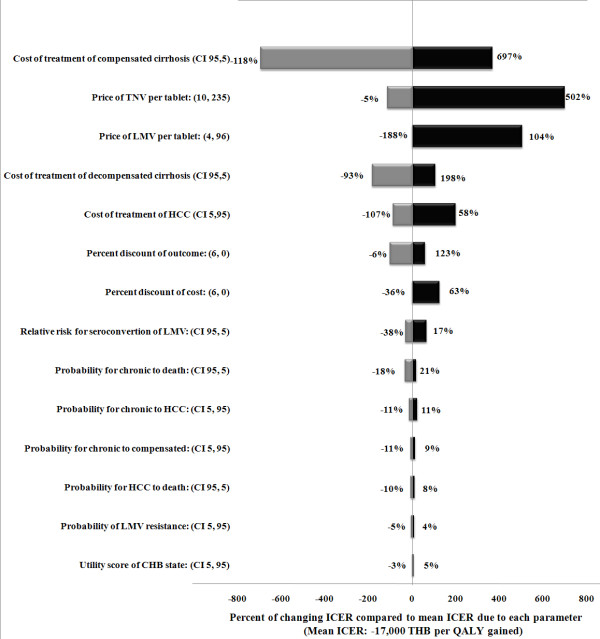
Tornado diagram illustrating the one-way sensitivity analysis results.

Figure 3 presents a tornado diagram illustrating the one-way sensitivity analysis results. Only the important parameters of the most cost-saving intervention were selected (i.e., generic lamivudine with the addition of tenofovir when drug resistance occurred). The outcome of this analysis showed which parameters the ICER per QALY gained were most sensitive to when altering the values, and they are listed as follows from most to least sensitive: the cost of treatment of compensated cirrhosis; the price of tenofovir; the price of lamivudine; the cost of treatment of decompensated cirrhosis; the cost of treatment of HCC; the discount rates of 0% and 6% per annum for cost and outcome; the relative risk of seroconversion of lamivudine; and the probability of transitioning from a CHB state to the death state.

**Figure 4:**
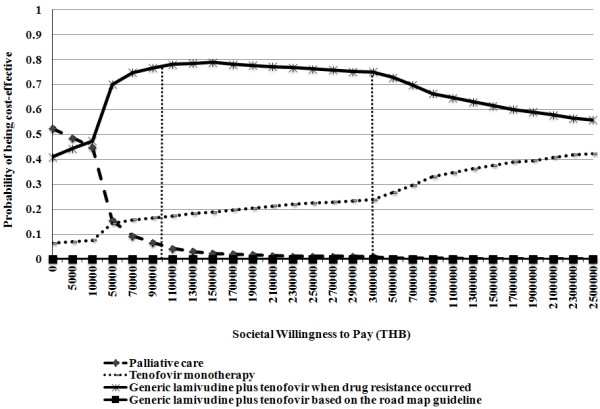
Cost-effectiveness acceptability curves presenting PSA results.

The PSA results are presented in Figure 4 using cost-effectiveness acceptability curves. To clearly present the results, other treatment alternatives were omitted except for tenofovir monotherapy, generic lamivudine with the addition of tenofovir when drug resistance occurred, and generic lamivudine with the addition of tenofovir based on the roadmap guideline compared with palliative care. The willingness to pay (WTP) threshold for one QALY for the adoption of health technologies and interventions is designated by the dashed vertical lines. At WTP thresholds of 100,000 (USD 3,333) and 300,000 THB (USD 10,000) per one QALY gained in Thailand, the probabilities for cost-effective treatment via the provision of generic lamivudine with the addition of tenofovir when drug resistance occurred were 78% and 75%, respectively. Moreover, the probabilities of tenofovir monotherapy being cost-effective were 18% and 24%, respectively. However, the probability of providing generic lamivudine with the addition of tenofovir based on the roadmap guideline being cost-effective was 0%, regardless of how much society was willing to pay for one QALY gained.

## Discussion

With the intent of aiding policy decision makers on which CHB drugs should be included in the NLED, our study was the first to compare the cost-utility of each drug therapy according to the THASL clinical practice guidelines with palliative care in patients with HBeAg-positive CHB based on a societal perspective. Even though the ICER results indicated that these three alternatives were dominant due to higher effectiveness and lower costs when compared with palliative care, it is evidenced that both tenofovir monotherapy and generic lamivudine with the addition of tenofovir when drug resistance occurred were more superior than generic lamivudine with tenofovir added based on the roadmap guideline when taking the uncertainty of all parameters in the model into account. The results have shown that the total cost of generic lamivudine with tenofovir added based on the roadmap guideline was higher compared with generic lamivudine plus tenofovir when drug resistance, whereas total LYs and QALYs obtained from both interventions were not different. As a result, when compared with providing generic lamivudine plus tenofovir added based on the road map guideline providing generic lamivudine plus tenofovir when drug resistance could save healthcare costs of approximately 70,000 THB (USD 2,333) per patient due to the cost avoidance of serious complications in the future. Thus, when considering the provision of CHB treatment to HBeAg-positive CHB patients above 30 years of age (i.e., 40-70 years), providing generic lamivudine plus tenofovir when drug resistance occurred and tenofovir monotherapy were dominant and cost-saving interventions compared with palliative care.

Furthermore, generic lamivudine, which has already been included in the NLED, should be considered as the first-line drug for the treatment of HBeAg-positive CHB patients above 30 years of age (i.e., 40-70 years) who require the treatment. In contrast to the findings of other previously published studies, entecavir [8-17], adefovir [6,7], telbivudine [5], and pegylated interferon [21,23,24] were more cost-effective compared with lamivudine. This could be explained by the fact that our study considered drug resistance due to lamivudine to imitate the real current clinical practice. Moreover, generic lamivudine, which is very inexpensive in Thailand, was also included as one of the interventions.

Although lamivudine can cause HBV DNA suppression in most HBeAg-positive CHB patients, it is also associated with a high rate of drug resistance [28]. Our study indicated that tenofovir, which was a cost-saving option, should be used as either the first- or second-line drug for the management of drug resistance due to nucleoside analog such as lamivudine. Similarly, the study of Buti et al. revealed that tenofovir was associated with lower costs and higher efficacy than entecavir, telbivudine, and adefovir [13]. At the time of the study, no other CHB treatments with low rates of drug resistance (e.g., tenofovir) had been included in the NLED yet, even though tenofovir demonstrated high antiviral efficacy and low drug resistance for patients with CHB [3,4]. Therefore, we submitted the cost-effectiveness information of CHB treatments along with policy recommendations to the Subcommittees for Development of NLED in May 2012 that tenofovir should be included in the NLED [56]. After the meeting, it was announced that tenofovir would be included in the NLED only for CHB patients with drug resistance due to nucleoside analog such as lamivudine under the condition that tenofovir should be used as an alternative and not as the first-line therapy.

However, based on the expert’s opinion, if both lamivudine and tenofovir were included in the NLED, using tenofovir as the first-line drug would be the better option given that tenofovir has a very low resistance rate. It would be more convenient for clinicians to provide tenofovir as the first-line treatment in order to reduce the time and cost of drug resistance management compared with providing lamivudine as the first-line drug. Even if the patients taking tenofovir developed drug resistance, lamivudine could be added later. It should be noted though that tenofovir has also been implicated in causing renal toxicity. Tenofovir can also cause acute renal failure, Fanconi syndrome, proteinuria or tubular necrosis. These side effects are due to accumulation of the drug in proximal tubules [57].

Moreover, three major issues (i.e., the prices of tenofovir and lamivudine, resistance rate of tenofovir, and costs of all complications and adverse drug events) need to be addressed. First, it was noted that the price of tenofovir in this study was obtained from the current market price of tenofovir in Thailand, which is relatively inexpensive due to the discounted price (43 THB or USD 1.43) proposed by the pharmaceutical company. At present, the price of tenofovir is approximately equal to that of entecavir in many countries. If the maximum expected price of tenofovir was assumed to be equal to the price of entecavir (235 THB or USD 7.83 per tablet), the ICER value would change from a dominant value to 100,000 THB (USD 3,333) per QALY gained when compared with palliative care. Similarly, if the price of lamivudine was adjusted to the price of original lamivudine, the ICER would be adjusted to 81,000 THB (USD 2,700) per QALY gained. Therefore, when changing the prices to current market prices, tenofovir monotherapy or lamivudine would still be cost-effective in the Thai context although they would not be cost-saving interventions [55].

Second, according to the current studies related to drug resistance, the resistance rate of tenofovir used in this study was 0% [28]. If the resistance rate of tenofovir was assumed to be equal to that of entecavir based on expert opinion, it would still be a cost-effective intervention in the Thai context with an ICER of 8,000 THB (USD 267) per a QALY gained compared with palliative care.

Third, the direct medical costs of complication states in this study were obtained from a published multi-center observational study of hepatitis C conducted at five major tertiary care hospitals in Thailand [48]. The costs of all complications (i.e., compensated cirrhosis, decompensated cirrhosis, and HCC) in patients with hepatitis C might be lower than those conducted in CHB patients. Based on the sensitivity analysis results, the costs of all complications had the greatest effect on the changes in ICER values. It is suggested that providing generic lamivudine plus tenofovir when drug resistance occurred and tenofovir monotherapy would be cost-effective options since the ICER values were lower than one times the Thai GDP per capita [55]. In addition, the costs of treatment of the adverse drug events were not considered in the study. In particularly, generic lamivudine may result in more adverse drug events or side-effects. However, it is expected that the adverse drug reactions of generic lamivudine and tenofovir may have little effect on an increase in the ICER values. Lok et al.’s study showed that lamivudine treatment had an excellent safety profile in HBeAg-positive CHB patients [58] and nausea was the only adverse event that occurred more frequently in CHB patients receiving tenofovir, which may cause little impact on the cost [59].

It is important that the limitations of this study need to be addressed. Due to the lack of transitional probabilities data for CHB patients in Thailand, some transitional probabilities were obtained from published articles in other countries [27,28,31-44]. However, we specifically performed a meta-analysis which could yield the most reliable evidence [30]. Furthermore, the utility data of CHB has been gathered but not for all complication states (i.e., compensated cirrhosis, decompensated cirrhosis, and HCC) in Thai CHB patients; therefore, the utility data of CHB patients with complications were obtained from a multinational study [54].

## Conclusions

Our results suggested that providing generic lamivudine with the addition of tenofovir when drug resistance occurred, generic lamivudine with the addition of tenofovir based on the roadmap concept, and tenofovir monotherapy were dominant and cost-saving interventions compared with palliative care. However, when taking into account all parameter uncertainties in the model, providing generic lamivudine with the addition of tenofovir when drug resistance occurred and tenofovir monotherapy would yield higher probabilities of being cost-effective at the societal WTP thresholds in Thailand compared with other alternatives. According to the cost-effectiveness results obtained from this study, the Subcommittees for Development of NLED decided to include tenofovir into the NLED. It is recommended that generic lamivudine should be used as the first-line drug and tenofovir should be considered when drug resistance occurs for HBeAg-positive CHB patients under the condition that tenofovir should be used as an alternative and not as the first-line therapy. Given that tenofovir is included in the NLED, a pharmaceutical reimbursement list of three health insurance schemes (i.e., Social Security Scheme, Civil Servant Medical Benefit Scheme and Universal Coverage Scheme) accounting for approximately 100% of Thai population, the cost of tenofovir incurred by all HBeAg-positive CHB patients under these health insurance schemes could be reimbursed by the Thai government. Consequently, this could definitely help HBeAg-positive CHB patients gain more access to tenofovir when drug resistance occurred and finally could improve patient outcomes and decrease mortality. However, due to a lack of information about drug resistance rate of tenofovir, it is also recommended that intensive monitoring and evaluation of drug resistance should continue to be performed by the Department of Disease Control and the Department of Medical Sciences.

## Abbreviations

HBV: Hepatitis B virus; HCC: Hepatocellular carcinoma; HBIG: Hepatitis B Immune Globulin; CHB: Chronic hepatitis B; FDA: Food and Drug Administration; NLED: National List of Essential Drugs; DMSIC: Drug and Medical Supply Information Center; CPI: Consumer price index; PPP: Purchasing power parity; SE: Standard Error; LYs: Life years; QALYs: Quality adjusted life years; THB: Thai baht; ICER: Incremental cost-effectiveness ratio; PSA: Probabilistic sensitivity analysis; NMB: Net monetary benefit; WTP: Willingness to pay; GDP: Gross domestic product; NHSO: National Health Security Office.

## Competing interests

The authors declare that they have no competing interests.

## Authors’ contributions

NT and UC performed the research, analyzed data, and drafted the manuscript. TT was involved in clinical research part and drafted the manuscript. PW participated in its design and drafted the manuscript. YT designed the research and drafted the manuscript. All authors read and approved the final manuscript.

## Pre-publication history

The pre-publication history for this paper can be accessed here:

http://www.biomedcentral.com/1472-6963/14/170/prepub
